# Sex Specific Associations of Sex hormone With Brain Volumes and Cerebral Blood Flow: A Cross Sectional Observational Study Within the Look AHEAD Type 2 Diabetes Cohort

**DOI:** 10.21203/rs.3.rs-4254188/v1

**Published:** 2024-04-29

**Authors:** Dhananjay Vaidya, Yvette Yeboah-Kordieh, Marjorie Howard, Christina E. Hugenschmidt, Paul A Nyquist, Erin D Michos, Rita R Kalyani, Sevil Yasar, Brian Andres Robusto, Hussein N Yassine, Jeanne M Clark, Mark A Espeland, Wendy L Bennett

**Affiliations:** Johns Hopkins University; University of Southern California; Wake Forest School of Medicine; Sticht Center for Healthy Aging and Alzheimer’s Prevention, Wake Forest School of Medicine; Johns Hopkins School of Medicine; Johns Hopkins University; Johns Hopkins University; Johns Hopkins University; Wake Forest University School of Medicine; University of Southern California; Johns Hopkins University; Wake Forest School of Medicine; Johns Hopkins University

## Abstract

**Background:**

Females have greater brain volume and cerebral blood flow than males when controlling for intracranial volume and age. Brain volume decreases after menopause, suggesting a role of sex hormones. We studied the association of sex hormones with brain volume, white matter hyperintensity volumes and cerebral blood flow in people with Type 2 Diabetes and with overweight and obesity conditions that accelerate brain atrophy.

**Methods:**

We analyzed data from 215 participants with overweight or obesity and Type 2 Diabetes from the Look AHEAD Brain Magnetic Resonance Imaging ancillary study (mean age 68 years, 73% postmenopausal female). Estradiol and total testosterone levels were measured with electrochemoluminescence assays. The ratio of brain measurements to intracranial volume was analyzed to account for body size. We analyzed sex hormones as quantitative measures in males, whereas in females we grouped those with detectable vs. undetectable hormone levels (Estradiol <73 pmol/L [20 pg/mL]: 79%; Total Testosterone < 0.07 mmol/L [0.02 ng/mL]: 37% undetectable in females).

**Results:**

Females with detectable total testosterone levels had higher brain volume to intracranial volume ratio (median [25^th^, 75^th^ percentile]: 0.85 [0.84, 0.86]) as compared to those with undetectable Total Testosterone levels (0.84 [0.83, 0.86]; rank sum p=0.04). This association was attenuated after age and body mass index adjustment (p=0.08). Neither white matter hyperintensity volumes or cerebral blood flow in females, nor any brain measures in males, were significantly associated with Estradiol or Total Testosterone.

**Conclusions:**

In postmenopausal females with Type 2 Diabetes with overweight and obesity, detectable levels of total testosterone were associated greater brain volume relative to intracranial volume, suggesting a protective role for testosterone in female brain health. Our findings are limited by a small sample size and low sensitivity of hormone assays. Our suggestive findings can be combined with future larger studies to assess clinically important differences.

**Trial Registration:**

NCT00017953

## Plain English Summary

Females have a larger relative size of the brain and blood flow than males after accounting for skull cavity size. Also, brain volume reduces after menopause, suggesting that sex hormones may affect brain size and health. This may be important in people with diabetes, a condition affecting brain health, but has not been well studied. In a smaller sample of 156 postmenopausal females and 59 similar-aged males within the Look AHEAD study of people with diabetes, we tested if levels of the sex hormones estradiol and testosterone were related to brain volumes, blood flow and the burden of early abnormalities known as white matter hyperintensities using Magnetic Resonance Imaging. We found no relationships between sex hormone levels and brain measures among males. Among postmenopausal females, brain volumes as a percentage of skull cavity volume was larger in females with detectable testosterone levels as compared to those without detectable hormone levels. This relationship was similar but not as strong when we took age and body mass index into account. Our results may be weaker than the actual relationship between sex hormones and brain volumes and health because our study sample was small, and the measurement of sex hormones was not sensitive enough for most postmenopausal females. However, our findings suggest a role for testosterone levels in preserving brain size in females and highlight the need for larger studies with sensitive hormone measurements to confirm and expand our results.

## Introduction

The volume of the brain progressively decreases during adult life but the decline is accelerated after 55 years of age, more so in older males.(1, 2) Because the brain depends primarily on glucose as an energy source,(3) changes in the brain may vary by age, sex and chronic diseases, particularly diabetes.(4) In the Look AHEAD (Action for Health in Diabetes) cohort of middle and older aged people with Type 2 Diabetes, there was lower prevalence of cognitive impairment, larger brain volumes and better cerebral blood flow (CBF) in postmenopausal females compared to similar-aged males.(5, 6) At the same time, in older females with Type 2 Diabetes, higher levels of estradiol levels and postmenopausal hormone therapy were associated with worsening cognitive function, increased risk of dementia and brain atrophy.(7, 8) These sex differences suggest a role for sex hormones in affecting brain volumes and blood flow that may qualitatively differ by sex.

However, very few studies have examined the association of sex hormones with brain volumes, blood flow and subclinical pathology detected as white matter hyperintensity (WMH) volumes and blood flow , as recently reviewed by Rehbein et al.(9) Importantly, no studies have specifically focused on sex hormones and brain health people with Type 2 Diabetes and overweight/obesity, a condition associated with accelerated brain atrophy and changes in cerebral blood flow. In addition, we do not know the association of brain health with menopausal timing in older females, which represents an integrated exposure to low estrogen, rather than menopause per se, which happens in in all women at older ages. In this analysis we leveraged an existing, well-characterized cohort of people living with Type 2 Diabetes who had been enrolled in the Look AHEAD trial(10) subsequently enrolled in a brain magnetic resonance imaging (MRI) ancillary study to assess the association of brain health and people aging with Type 2 Diabetes with obesity and overweight. Our main aim was to investigate the association of sex hormones. i.e., estradiol and testosterone levels, in both sexes and of time since menopause in females, with brain volumes, white matter hyperintensity volumes and cerebral blood flow in middle to older-aged individuals living with Type 2 Diabetes with obesity and overweight.

## Methods

### Study population

The Look AHEAD study was a multisite (16 sites), single blind randomized control trial that recruited 5,145 participants from the age of 45–74 years diagnosed with Type 2 Diabetes with obesity and overweight to assess the impact of lifestyle intervention on cardiovascular disease (main outcome), diabetes control and other cardiometabolic outcomes.(10) Participants were randomized to an Intensive Lifestyle Intervention (ILI) or a Diabetes Support and Education (DSE) group. The ILI targeted reducing caloric intake and increasing physical activity to induce weight loss >7% and maintaining this over time. DSE participants were invited to attend group sessions focused on diet, physical activity, and social support.(11, 12)

**Figure 1:**
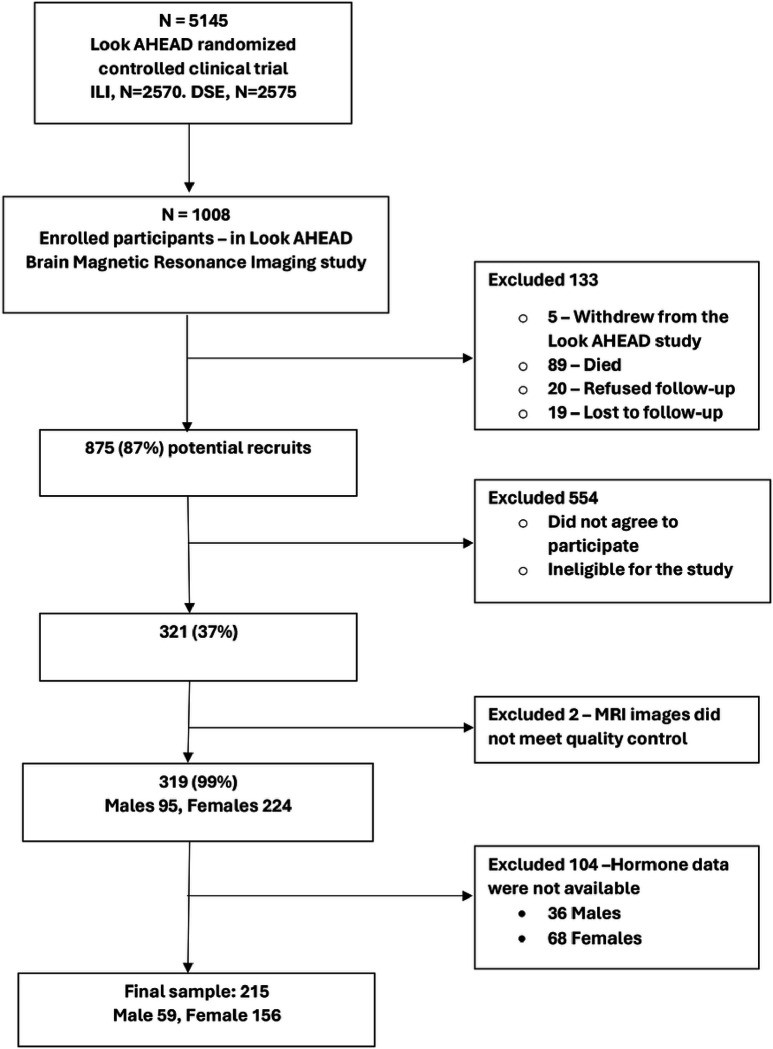
Flow Chart of Sample Selection and Exclusions

To be included in the study, the participants needed to have overweight with a body mass index (BMI) of at least 25kg/m^2^ or 27kg/m^2^ if currently taking insulin.(10) After the initial intervention phase of the trial period of 8 years (ranging from 8 to 11 years among participants), the study continued as an observational cohort. 1008 participants were enrolled in an ancillary study, the Look AHEAD Brain study, which aimed to use MRI to evaluate brain structure and function once at 10–12 years from the original enrollment date.(6) When available, a serum sample collected between years 8–13 years of original enrollment, i.e., as close in time as possible to the date of MRI, was chosen for the analysis of sex hormone levels. For this analysis we selected males and females who had both measured sex hormones and a brain MRI. Figure 1 shows the sequential inclusion/exclusion flow of the sample included in our analysis, with a final sample size of 156 females and 59 males.

### Brain MRI

As described in other reports,(13) MRI imaging was performed using 3.0T scanners (Siemens, Phillips, GE) running similar pulse sequences on all scanners. Multiatlas label fusion method was used to partition the brain to allow volumetric measurements at various resolutions. We used standard methods to identify normal brain volume, volume of abnormal white matter hyperintensities and cerebral blood flow. The ancillary study computed volumes for the whole brain, gray matter, white matter, and the ventricles for each study participant. To control for head size, we adjusted for total intracranial volumes (ICVs). White matter Hyperintensity volumes were segmented using a supervised learning-based multimodal segmentation method .(14) Cerebral blood flow was assessed with multi-phase pseudo-Continuous Arterial Spin Labeling with background suppression for labeling the endogenous blood water.(6) For the main analyses, we examined total brain volume, total volume and total cerebral blood flow. A list of regional measures for volume and blood flow is shown in supplementary tables S1-S4.

### Estradiol and testosterone levels

Sex hormone assays were run on one available serum sample per individual collected between years 8–13. Estradiol and testosterone levels were estimated with Roche reagent Elecsys Estradiol III and Elecsys Testosterone II respectively on a Cobas e801 Immuno-analyzer. The analyzers used two monoclonal antibodies specifically directed against 17β-estradiol and a monoclonal antibody specifically directed to testosterone.

### Statistical Analysis:

All statistical analyses were performed separately for female and male samples. We tabulated demographic and clinical characteristics as numbers and percentages for categorical variables, means and standard deviations for symmetrically distributed continuous variables and medians and 25^th^ to 75^th^ percentile ranges for other continuous variables. We explored the extent to which hormone levels were high enough to be detectable in each of the sexes. For the female stratum, 80% of samples had lower than quantifiable levels of estradiol and 37% had lower than quantifiable levels of testosterone in the immunoassay. Thus, for the female stratum, all hormone level analyses were performed comparing the dichotomous variables of detectable versus undetectable hormone levels. Age at menopause in females was analyzed as a continuous variable. In males, hormones were analyzed quantitatively. All male participants had detectable testosterone levels, and 9 participants had undetectable estradiol levels. For analysis of hormone levels as continuous variables in males, we imputed the value of 0.5*the minimum measured hormone level for those with undetectable hormone levels.(15) For unadjusted non-parametric analyses, undetectable levels were assigned ranks below detectable levels. For regression analyses, we imputed the values as 0.5× (detection limit of the assay). Because the hormone levels were right-skewed, they were logarithmically transformed prior to regression analyses. Prior to regression results, we tabulated medians and interquartile ratios of brain volumes with ICV groups by detectable vs undetectable hormone levels in females or by Spearman correlations of the ratios with hormone levels in males.

For regression analyses, hormone levels and white matter hyperintensity volumes were logarithmically transformed because they were right-skewed. All models were adjusted for ICV. Based on exploratory bivariable correlation along with causal reasoning regarding confounding, we also analyzed a model further adjusted for age and BMI as prespecified covariates. Other covariates explored included original treatment allocation to lifestyle intervention, Hemoglobin A1c percent, systolic blood pressure, total cholesterol levels. Any explored covariate associated with both a brain measurement and a hormone measurement was included as a covariate in the regression analysis for that specific brain variable on that specific hormone variable and results regarding change qualitative change in the brain-hormone association were noted.

For the regional brain data in supplementary analyses, given the exploratory nature of the associations with many p-values calculated, we have presented the point estimates of association, and standard errors data for possible metanalyses with future studies but do not interpret the p-values for statistical significance.

Analyses were conducted using R statistical software (Version 4.2.3, implemented in RStudio Version 2023.03.0+386), with additional sensitivity analyses and graphic production performed using Stata (Version 18, Statacorp, College Station, TX).

## Results

### Characteristics of the study population:

Table 1 shows the demographic and clinical characteristics of the study sample. The sample was predominantly female (73%). 50% of the females in the sample had been randomized to ILI (Intensive Lifestyle Intervention) and, 58% of males had been randomized to ILI. The age distribution in both sexes was similar with a mean age of 68 years, and the racial distribution was predominantly White (85% among males and 71% among females). A larger proportion of males had >16 years of education (64% for males vs. 37% for females). The clinical characteristics in both females and males reflected the source population for the parent study of people with overweight or obesity and Type 2 Diabetes, with mean systolic blood pressure in the hypertensive range and HbA1c in the diabetes range. Among males, testosterone was detectable in all samples, and estradiol was undetectable in 15% of the samples. Among females, testosterone was undetectable in 37% of samples and E2 was undetectable in 80% of the samples.

### Association of sex hormones with brain measures-to-intracranial volume ratios:

Table 2 shows that in females, there were no differences in the ratio of brain measures to ICV by detectable (vs. undetectable) levels of estradiol. Females with detectable levels of testosterone had larger total brain volumes as a ratio to ICV as compared to those without detectable levels of testosterone, with the distributions shown in Figure 1. As also shown in Table 2, the associations with white matter hyperintensity volumes and cerebral blood flow were not statistically significant. After adjustment for age and BMI, none of the associations were statistically significant. Detailed associations of regional brain volumes with sex hormones in females are shown unadjusted in Supplementary Table S1 and adjusted in Supplementary Table S2. The significant unadjusted positive association of total brain volume with detectable testosterone levels was mirrored by a negative association with ventricle volume and a positive association with frontal lobe volume. Age at menopause was not statistically significantly associated with either sex hormone (Supplementary Table S3).

As seen in Table 3, in males, there were no statistically significant associations between total brain volume or white matter hyperintensity volume or cerebral blood flow and either sex hormone. Detailed associations of regional brain volumes with sex hormones in males are shown unadjusted in Supplementary Table S4 and adjusted in Supplementary Table S5.

The supplementary material also provides the standard deviations of all variables by their sex stratum for the purpose of interpreting standardized associations.

Sensitivity analyses: Among exploratory covariates, none were identified as possible confounders that necessitated inclusion into adjusted models.

## Discussion

This is one of the first studies to assess associations between sex hormones and brain volume in people living with Type 2 Diabetes. In our study we have found that in females with Type 2 Diabetes and with obesity and overweight, higher levels of testosterone were associated with larger brain volumes after adjusting for covariates. However, we did not find statistically significant associations between total brain volume, white matter hyperintensity volume, or cerebral blood flow and estradiol levels or age at menopause in females or sex hormones in males.

Our exploratory regional brain analysis may either be combined or serve as a reference for future studies that localize sex hormone associations within the brain regions. While Type 2 Diabetes is generally associated with more white matter hyperintensity,(16) the role of sex hormones in persons with Type 2 Diabetes had not previously been studied. In another special population, women living with HIV had a trend to positive association of testosterone with total white matter volume, and more so with another androgenic hormone (dehydroepiandrostenediane sulphate).(17) To the extent that people with HIV are also in metabolic stress, this pattern is consistent with our results. Total testosterone may be a proxy for its tightly bound carrier, Sex Hormone Binding Globulin, shown to be associated with greater white matter volumes.(18)

A review of the association of sex hormones with brain volume measures in females conducted by Rehbein and colleagues.(9) showed inconsistent findings across studies; some reports showed positive associations while others were negative. Also in males, we found inconsistent positive and negative of associations of sex hormones with regarding brain volumes.(18, 19) Some studies examined the association of sex hormones with early subclinical brain disease represented by white matter hyperintensity volumes in brain MRIs with estrone but not estradiol species longitudinally associated with greater WMH in women,(20) and testosterone not being associated with white matter hyperintensity volumes in men.(21)

Possible mechanisms for the brain protection by testosterone may be immunomodulation as reviewed by Whitacre et al.(22) as well as increase in neurotrophic factors as reviewed by Bialek et al.(23) as has been shown in animal models. Although it is difficult to directly relate these animal models directly to aging humans with diabetes, testosterone treatment in postmenopausal women has been associate with learning and memory consistent with a neuroprotective effect.(24)

Our study has several strengths. Because the sample was nested within the Look AHEAD study, clinical and Type 2 Diabetes and obesity and overweight related measures are well characterized for approximately a decade. Study participants underwent detailed MRI evaluation with assessment of total and regional measures or brain volume, white matter hyperintensity volumes and cerebral blood flow.

Our study also has several important limitations. The follow-up cohort from a past clinical trial is observational in design and has the limitations inherent to such studies, and we cannot account for unknown or unmeasured confounders nor make assumptions regarding causality. First, the sub-analysis was restricted to a non-random, convenience sample of individuals with both MRI and blood samples, which can introduce the bias of retention of healthy participants. Second, similar to some large prior studies,(25) the measurement of hormone levels by chemoimmunoassay did not have adequate sensitivity to measure hormone levels in a large proportion of postmenopausal females, restricting the comparison to detectable vs. undetectable levels; Third, Sex Hormone Binding Globulin was not measured in the samples to determine the extent to which testosterone was bound, free of bioavailable. Fourth, initially enrolled for a randomized clinical trial, our study population was highly educated and predominantly of White race, limiting its generalizability.

In conclusion, our study suggests that in postmenopausal females with Type 2 Diabetes and with obesity and overweight, higher (i.e., detectable) testosterone levels were associated with greater preservation of brain volume, but no association with white matter hyperintensity volumes. Our results also bring into focus the need for more studies in people living with Type 2 Diabetes with obesity and overweight, using high sensitivity hormone assays to measure hormones in postmenopausal females. In addition, future studies need to disaggregate testosterone levels into bioavailable testosterone levels and versus those associated with Sex Hormone Binding Globulin. Our study provides extensive exploratory results by brain volume regions that may be combined with future research studies assessing the relationship between brain volume, cerebral blood flow and sex hormones.

## Figures and Tables

**Figure 2:**
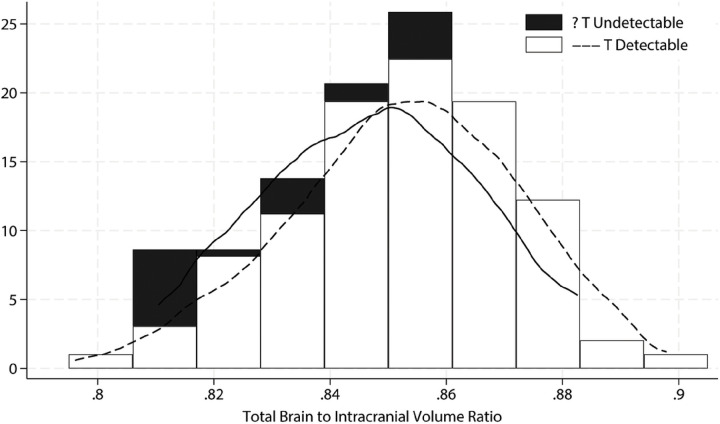
Histogram of Total Brain to Intracranial Volume Ratio by Detectable vs. Undetectable Testosterone Levels in Females, with Overlaid Kernel density Smooth Curves.

**Table 1:** Demographic and Clinical Characteristics of the Study Sample

	Male	Female
	N=59	N=156
**Intervention group**		
DSE	25 (42%)	78 (50%)
ILI	34 (58%)	78 (50%)
**Age**	68.3 [65.5, 71.4]	68.6 [64.6, 73.7]
**Age at menopause**	NA	50 [40, 52]
**Race/Ethnicity**		
Black	6 (10%)	39 (25%)
Native American	0 (0%)	1 (1 %)
Asian	0 (0%)	2 (1 %)
White	50 (85%)	110 (71 %)
Hispanic	3 (5%)	2 (1 %)
Other	0 (0%)	2 (1 %)
Education *		
<13 years	4 (7%)	26 (17%)
13–16 years	15 (25%)	65 (42%)
>16 years	38 (64%)	57 (37%)
**BMI**	32.4 [30.1,35.5]	33.3 [29.7, 37.6]
**Current smoker**	1 (2%)	3 (2%)
**Waist circumference**	113 [106, 123]	108 [100, 116]
**Systolic blood pressure**	132 [115, 138]	130 [114, 141]
**Diastolic blood pressure**	70 [65, 74]	67 [59, 72]
**Total cholesterol**	152 [134, 168]	167 [147, 195]
**Self-reported duration of diabetes**	5 [2, 10]	5 [2, 7]
**HbA1c**	6.9 [6.2, 8.2]	7.1 [6.4, 8.2]
**Medications**		
Insulins	11 (17%)	40 (26%)
Lipid Lowering Medications	45 (76%)	120 (77%)
Antihypertensive Medications	50 (85%)	119 (76%)
**Estradiol in pmol/L**	103 [81, 127]	<73 [<73, <73]
**Undetectable estrogen levels (<73 pmol/L)**	9 (15%)	124 (80%)
**Testosterone in nmol/L**	12.8 [10.6, 18.6]	0.24 [<0.07, 0.49]
**Undetectable testosterone levels in (<0.07 nmol/L)**	0 (0%)	58 (37%)

Tabulated as number (percentage) for categorical variables, median [25^th^, 75^th^ percentile] for continuous variables,

* Education percentages do not add to 100% due to a small number of missing values

**Table 2:** Associations of the ratios of Brain Measures With Circulating Hormone Levels in Females

	Unadjusted, Ratio/ICV			Adjusted for ICV, age and BMI	
	Hormone Undetectable	Hormone Detectable	Rank Sum p-value	Standardized β ± SE	p-value
	**Estradiol**				
**Total Brain**	0.85 [0.83, 0.86]	0.85 [0.84, 0.86]	0.42	0.06 ± 0.05	0.22
**WMH**	1.7 [0.7, 4.3]×10^−3^	1.6 [0.9, 3] ×10^−3^	0.72	−0.17 ± 0.19	0.36
**Total CBF**	38.0 [32.5, 46.3] ×10^−3^	36.9 [33.4, 43.2] ×10^−3^	0.65	−0.09 ± 0.2	0.66
	**Testosterone**				
**Total Brain**	0.84 [0.83, 0.86]	0.85 [0.84, 0.86]	0.04	0.08	0.55
**WMH**	1.4 [0.7, 4.3] ×10^−3^	1.7 [0.9, 4.2] ×10^−3^	0.62	−0.14	0.30
**Total CBF**	38.3 [31.8, 46.5] ×10^−3^	37.6 [32.8, 46.1] ×10^−3^	0.98	−0.13	0.32

Ratios tabulated as median [25^th^, 75^th^ percentile]; ICV - intracranial volume; WMH - White Matter Hyperintensity Volume; CBF - Cerebral Blood Flow; Standardized β - SD Brain Measure/Detectable vs. Undetectable Hormone

**Table 3:** Associations of the ratios of Brain Measures With Circulating Hormone Levels in Males

	Unadjusted, Ratio/ICV		Adjusted for ICV, age and BMI	
	Spearman Correlation	p-value	Standardized β ± SE	p-value
	Estradiol			
**Total Brain**	0.06 ± 0.05	0.22	0.03 ± 0.05	0.48
**log_e_(Total WMH)**	−0.17 ± 0.19	0.36	0.06 ± 0.13	0.64
**Total CBF**	−0.09 ± 0.2	0.66	0.05 ± 0.14	0.74
	**Testosterone**			
**Total Brain**	0.08 ± 0.04	0.08	0.06 ± 0.05	0.21
**log_e_(Total WMH)**	0.01 ± 0.16	0.93	−0.16 ± 0.14	0.25
**Total CBF**	0.06 ± 0.17	0.74	−0.17 ± 0.15	0.27

ICV - intracranial volume; WMH - White Matter Hyperintensity Volume; CBF - Cerebral Blood Flow; Standardized β - SD Brain Measure/SD of log_e_(Hormone)

## Data Availability

Data and biospecimens from the Look AHEAD Study can be obtained from the NIDDK central repository at: https://repository.niddk.nih.gov/studies/look-ahead/

## Highlights

- Females have larger brain sizes as a fraction of intracranial volume than similar aged males, and lose brain tissue after menopause, suggesting a role for sex hormones in brain size and health. Although this relationship may be important in the brain health of older people living with diabetes, it has not been well-studied.
- In a study nested within the long-term observation period of the Look AHEAD clinical trial, postmenopausal women with detectable testosterone levels had larger brain volumes as compared to those without detectable testosterone levels. In these analyses there were no other associations.
- Study results may be limited by a small sample size and older, less sensitive hormone assays.
- Future larger studies with sensitive assays should examine and confirm associations of sex hormones with brain health.

## Abbreviations

CBF: Cerebral Blood Flow
DSE: Diabetes Support and Education
ICV: Intracranial Volume
ILI: Intensive Lifestyle Intervention
MRI: Magnetic Resonance Imaging
WMH: White Matter Hyperintensity
